# In vivo calibration of the T2* cardiovascular magnetic resonance method at 1.5 T for estimation of cardiac iron in a minipig model of transfusional iron overload

**DOI:** 10.1186/s12968-021-00715-6

**Published:** 2021-03-11

**Authors:** Peter Diedrich Jensen, Asbjørn Haaning Nielsen, Carsten Wiberg Simonsen, Ulrik Thorngren Baandrup, Svend Eggert Jensen, Martin Bøgsted, Sigridur Olga Magnusdottir, Anne Birthe Helweg Jensen, Benedict Kjaergaard

**Affiliations:** 1 Department of Hematology, Aalborg University Hospital, PO box 365, 9100 Aalborg, Denmark; 2 Department of the Built Environment, Aalborg University, Aalborg, Denmark; 3 Department of Radiology, Aalborg University, Aalborg, Denmark; 4 Centre for Clinical Research, North Denmark Regional Hospital, Hjoerring, Aalborg University Hospital, Aalborg, Denmark; 5 Department of Cardiology, Aalborg University Hospital, Aalborg, Denmark; 6 Department of Clinical Medicine, Aalborg University Hospital, Aalborg, Denmark; 7 Biomedical Research Laboratory, Aalborg University Hospital, Aalborg, Denmark; 8 Department of Cardiothoracic Surgery, Aalborg University Hospital, Aalborg, Denmark; 9 The Faculty of Medicine, Aalborg University Hospital, Aalborg, Denmark

**Keywords:** T2* cardiovascular magnetic resonance, Cardiac iron, T2* calibration, Minipig model, Dextran-iron loading, Cardiosiderosis

## Abstract

**Background:**

Non-invasive estimation of the cardiac iron concentration (CIC) by T2* cardiovascular magnetic resonance (CMR) has been validated repeatedly and is in widespread clinical use. However, calibration data are limited, and mostly from post-mortem studies. In the present study, we performed an in vivo calibration in a dextran-iron loaded minipig model.

**Methods:**

R2* (= 1/T2*) was assessed in vivo by 1.5 T CMR in the cardiac septum. Chemical CIC was assessed by inductively coupled plasma-optical emission spectroscopy in endomyocardial catheter biopsies (EMBs) from cardiac septum taken during follow up of 11 minipigs on dextran-iron loading, and also in full-wall biopsies from cardiac septum, taken post-mortem in another 16  minipigs, after completed iron loading*.*

**Results:**

A strong correlation could be demonstrated between chemical CIC in 55 EMBs and parallel cardiac T2* (Spearman rank correlation coefficient 0.72, P < 0.001). Regression analysis led to [CIC] = (R2* − 17.16)/41.12 for the calibration equation with CIC in mg/g dry weight and R2* in Hz. An even stronger correlation was found, when chemical CIC was measured by full-wall biopsies from cardiac septum, taken immediately after euthanasia, in connection with the last CMR session after finished iron loading (Spearman rank correlation coefficient 0.95 (P < 0.001). Regression analysis led to the calibration equation [CIC] = (R2* − 17.2)/31.8.

**Conclusions:**

Calibration of cardiac T2* by EMBs is possible in the minipig model but is less accurate than by full-wall biopsies. Likely explanations are sampling error, variable content of non-iron containing tissue and smaller biopsies, when using catheter biopsies. The results further validate the CMR T2* technique for estimation of cardiac iron in conditions with iron overload and add to the limited calibration data published earlier.

## Background

Cardiosiderosis is a serious complication of treating patients with chronic anaemias with blood transfusions, as for example in thalassaemia major [1, 2]. If iron chelation is insufficient, this can lead to myocardial dysfunction, heart failure and prematuredeath. The cardiac iron concentration (CIC) cannot be accurately predicted from body-iron loading markers [3], and unfortunately, ventricular dysfunction, as a measure of cardiosiderosis, is not useful, because it is a late, but serious finding [4]. Thus, early detection of cardiosiderosis is essential. In a few earlier studies, endomyocardial catheter biopsies (EMBs) have been used to assess the CIC [5–8]. Although EMB has proved its value in several fields of cardiology [9], this procedure is not routinely in use for assessment of cardiac iron due to its risk of morbidity and mortality.

Today, non-invasive estimation of CIC by cardiovascular magnetic resonance (CMR) is the preferred method. Different CMR-derived, iron-sensitive parameters have been investigated, such as T1 and T2 [10, 11], T2* [3, 12] and also the signal-intensity ratio between myocardium and skeletal muscle [13, 14]. Measurement of the relaxation parameter T2* has been shown as the most reliable and reproducible method for estimating myocardial iron loading [15–17]. T2* is predictive of cardiac complications [18], probably even better than indices of cardiac function [19].

While the clinical value of the T2* method has been well validated, studies calibrating cardiac T2* to chemically measured CIC are few [11, 12, 20] and EMBs have not been used. In two studies, human myocardial tissue became available for calibration due to special circumstances [12, 20]. In the study of Ghugre et al. [20], cardiac tissue from one thalassaemia major patient with transfusional iron overload (TIO), who had died from sepsis, was used. CMR was performed post-mortem. That study was the first to demonstrate that cardiac R2* is predominantly determined by CIC in patients with cardiosiderosis. Determination of the relationship between R2* and CIC was based on comparison of regional variation in R2* with regional iron concentration in the heart. Accordingly, the range of CIC and R2* values were small, and the calibration curve was limited to a narrow range. In the study of Carpenter et al*.* [12], biopsies came from 12 human formalin-fixed hearts, gathered by international collaboration, from transfusion-dependent patients with end-stage heart failure following death or cardiac transplantation. Cardiac T2* was measured after slicing the formalin-fixed hearts.

In the study of Wood et al. [11] the Mongolian gerbil model of TIO was used for calibration of cardiac T2*. The use of iron-loaded animals enabled in vivo measurement of cardiac T2*. Collectively, the 2 studies of Carpenter et al*.* [12] and Wood et al*.* [11] showed close correlations between cardiac T2* values and corresponding chemical CIC measurements in the cardiac septum, within the clinically relevant range of CIC, but slope difference between calibration curves was evident. Possible explanations are the use of ex vivo scanning of fixed, human hearts in one, and in vivo measurement of cardiac T2* in an animal model in the other study. Another possible explanation for the outcome of the Mongolian Gerbil study might be the hardware constraints, imposed by the use of a clinical CMR unit for scanning of small animals.

The main purpose of the present study was to perform a calibration of cardiac T2*, measured by in vivo CMR, with chemical CIC measurements on EMBs from right ventricular septum taken in parallel. For this purpose, we used a recently described minipig model of TIO, based on long-term parenteral dextran-iron loading [21]. This model enables simultaneous follow up with multiply repeated EMBs and clinical CMR for measurement of cardiac T2*. As sampling error may be an issue when using catheter biopsies for assessment of CIC, we also wanted to investigate, if better calibration of cardiac T2* is achieved by use of full-wall biopsy samples from cardiac septum, for chemical CIC measurement, taken immediately after euthanasia from the pig, after finished dextran-iron loading.

## Material and methods

### Animals, care and iron-loading

**Fig. 1:**
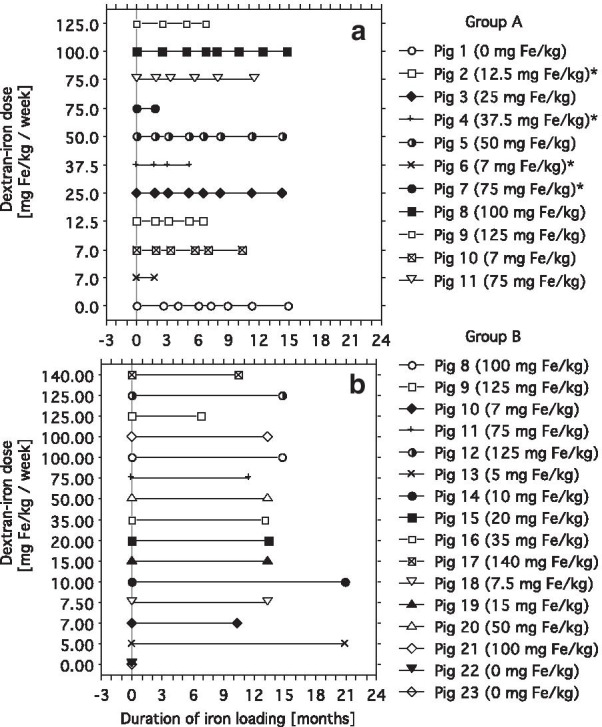
Individual pig timelines of dextran-iron dosing and timepoints of investigations. **a** Illustrates for each pig in group A the weekly intramuscular dosing of dextran iron, given in mg Fe/kg BW/week, the duration of iron loading in months and the timepoints of cardiovascular magnetic resonance (CMR) and of endomyocardial biopsies (EMB), performed at the end of the sessions. The dose of dextran iron in mg Fe/kg/ week was fixed in all pigs during the whole follow-up, but the total weekly dose injected was adjusted to the body weight (BW) immediately after each CMR/biopsy session. Pig 2, 4, 6 and 7 died of biopsy complications, indicated by * (for details see the *Results*). **b** Illustrates the dextran-iron dosing and duration of iron loading of the  pigs in group B, comprising 16  pigs, who had taken a full-wall biopsy from the cardiac septum immediately after last CMR session followed by euthanasia. Group B also includes pig 8 to 11 from group A, who also had these biopsies taken after euthanasia. Principles of iron loading are identical in both groups, except in pig 13 and 14: The dextran-iron dose was increased from 5 and 10 mg Fe/g, respectively, to 35 mg/kg in both pigs after about 12 months of iron loading

**Fig. 2:**
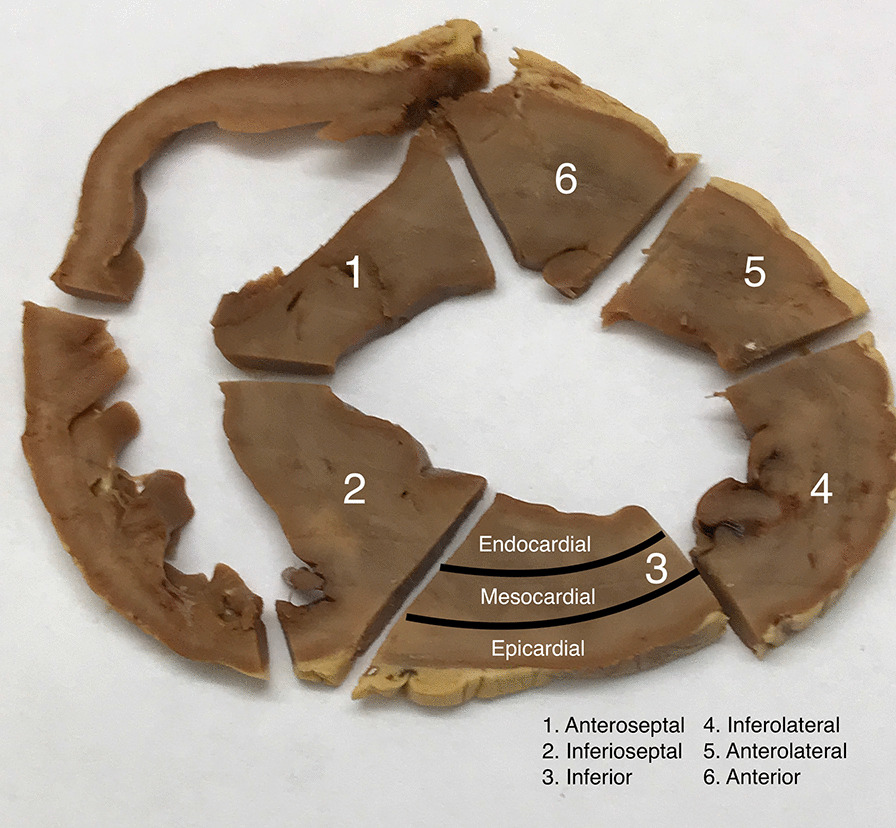
Investigation of segmental and transmural myocardial iron distribution. The photograph shows a mid-myocardial cut slice from the formalin-fixed heart of pig 8. The slice was cut into 6 segments: Anteroseptal (1), inferolateral (2), inferior (3), inferoseptal (4), anterolateral (5) and anterior (6). Each segment was divided into an epicardial-, mesocardial and endocardial layer (as indicated for segment 3), leading to 18 tissue samples from each heart for chemical cardiac iron concentration (CIC) assessment. Epicardial layers of the right ventricular septum (segment 1 and 2) represent endocardium

**Fig. 3:**
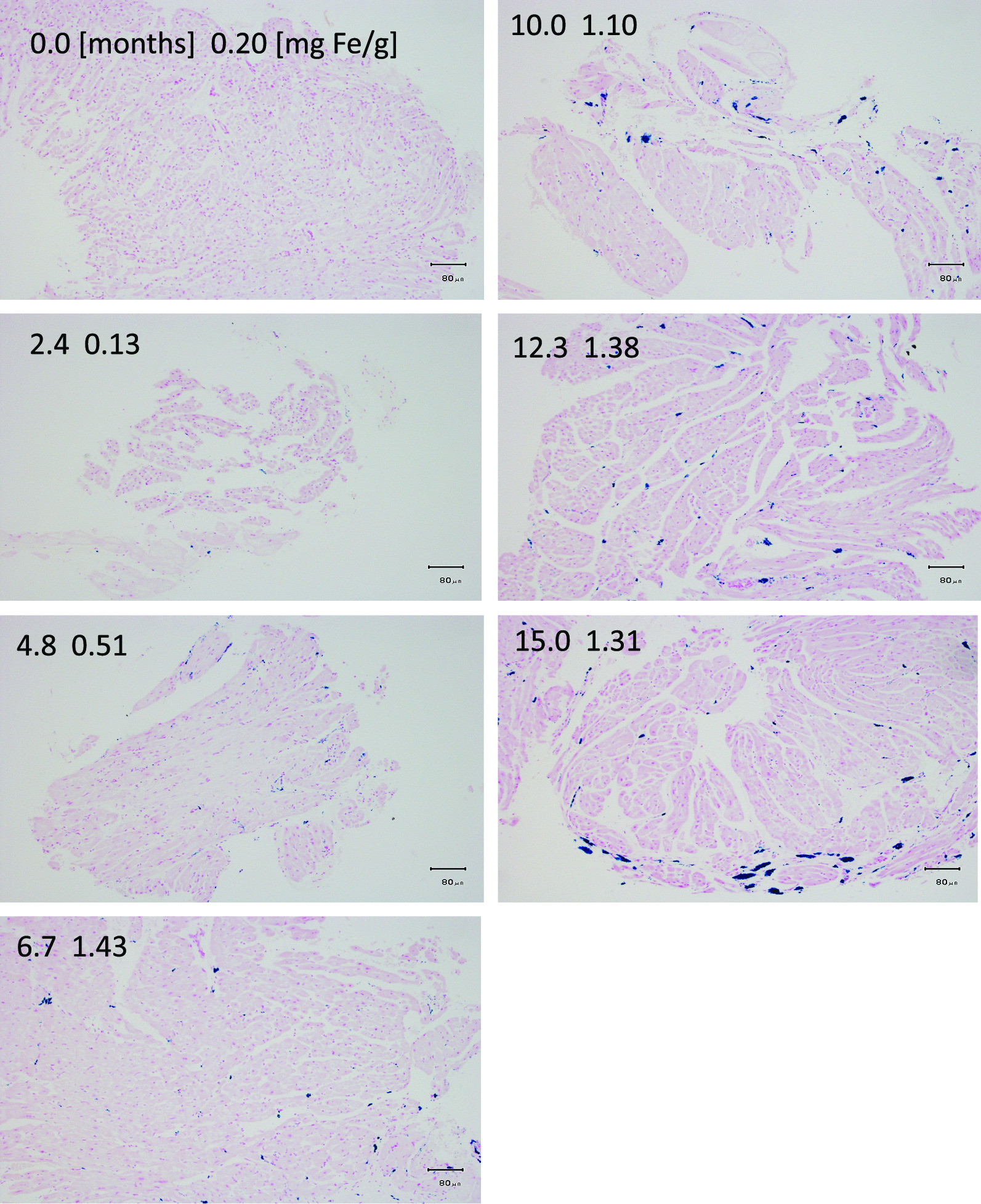
Color microphotograph of iron stains of sections from cardiac septum during iron loading. Light photomicrographs of endomyocardial right-ventricular catheter-biopsy sections taken during follow up of iron-dextran loading in pig 8, having received weekly, intramuscular dextran-iron injections, corresponding to 100 mg Fe/kg, for 15 months. Sections were stained using Perls’ Prussian blue reaction to demonstrate intracellular iron. Upper left corner in each panel indicates the month of iron loading when the biopsy was taken (left value) and contemporary chemical cardiac iron concentration (CIC) in mg/g (right value). Scale bar is 80 µm

**Fig. 4:**
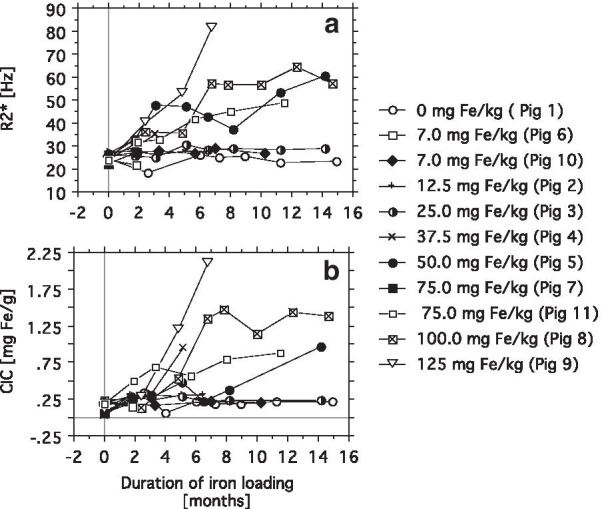
Simultaneous follow up of cardiac R2* and chemical CIC by EMBs during iron loading. Cardiac T2* (**a**) and chemical CIC (**b**) were followed up with 1–3 months intervals during dextran-iron loading in 11 minipigs for up to 15 months.  Pigs 2, 4, 6, 7 died due to biopsy-related complications after 6.5, 5, 2 and 2 months of iron loading, respectively. In pig 4, parallel 5-months results of CIC and R2* were not available. Chemical CIC was assessed by ICP in EMBs, taken from cardiac septum in connection to the CMR sessions. For each  pig, the weekly applied dextran-iron dose is indicated within the figure label as mg/kg/BW

**Fig. 5:**
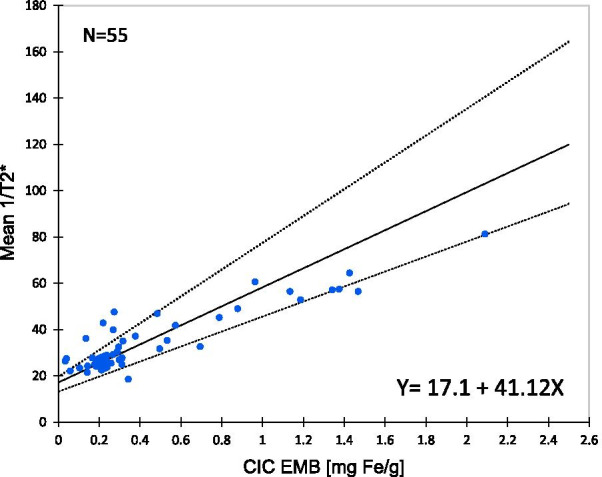
Calibration of cardiac R2* with CIC assessed in EMBs. The figure displays the relationship of cardiac R2* (= 1/T2*) to CIC. Non-parametric regression analysis (Passing and Bablok) led to the calibration equation [CIC] = (R2* − 17.16/41.12 with CIC in mg/g DW and R2* in Hz. The total number of parallel cardiac R2* and chemical CIC measurements was 55. Solid line is the regression line and dotted lines display its confidence interval. ULN for cardiac R2* is 28.18 Hz and ULN for CIC 0.31 mg Fe/g DW

**Fig. 6:**
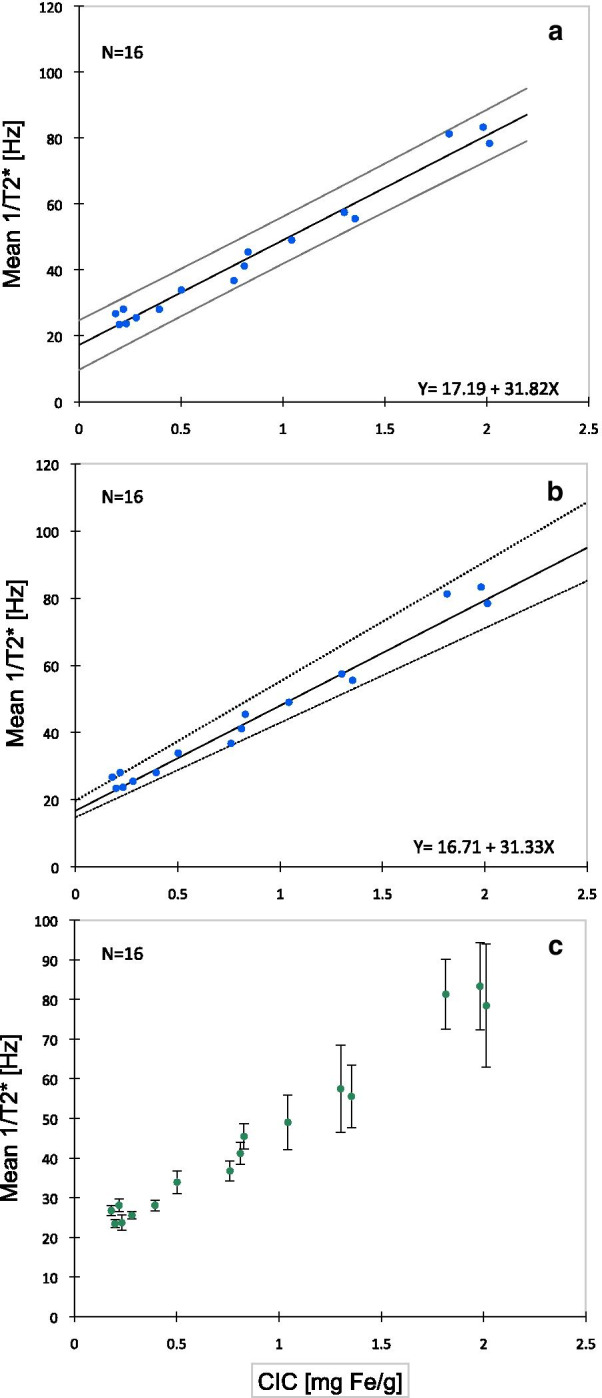
Calibration of cardiac T2* with full-wall biopsies. Full-wall biopsies were taken from cardiac septum (anteroseptal region) from 16  minipigs for assessment of chemical CIC by ICP. Biopsies were taken immediately after final CMR session, performed after completed iron loading. Fourteen of the 16 pigs had been dextran-iron loaded from 10.5 to 22 months by weekly intramuscular dextran-iron injections, and 2 had not (control  pig). Weekly dextran-iron dose was from 5 to 140 mg Fe/kg BW. **a** Displays the relationship of cardiac R2* to chemical CIC, investigated by classical linear regression analysis, leading to the calibration equation [CIC] = (R2* −  17.19)/31.82 with CIC in mg/g DW and R2* in Hz. In **b** non-parametric regression analysis was used (Passing and Bablok), leading to the calibration equation [CIC] = (R2* − 16.71)/31.33. Solid lines represent the regression lines and dotted lines display their confidence interval. **c** Displays the same data as in panel A and B, but the individual cardiac R2* measurements are given with error bars (mean of triplicate measurements ± SD)

**Fig. 7:**
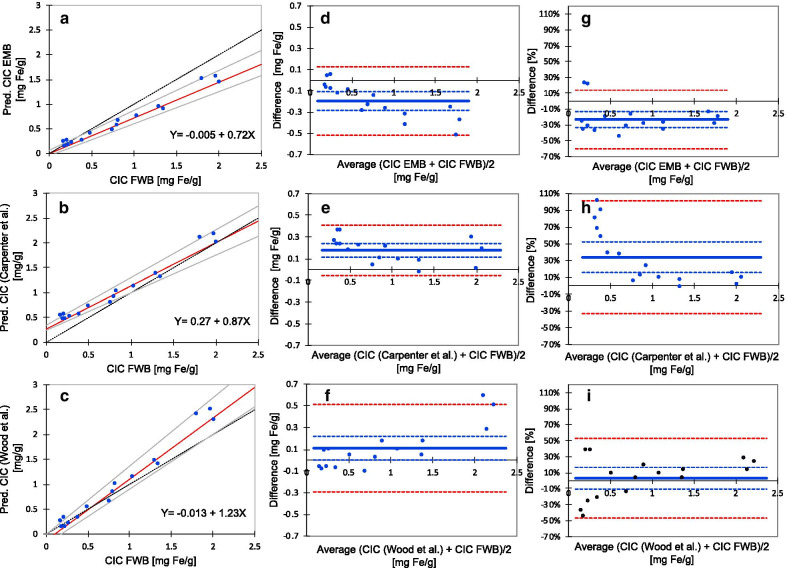
Comparison of chemical CIC measurements with T2*-based CIC estimates using 3 different calibration curves. **a**–**c** Display the relationship between CIC values, assessed chemically by use of post-mortem full-wall biopsies from cardiac septum (independent variable) and the corresponding CIC values predicted by the T2* method (dependent variable) using different calibration curves. In **a**, CIC values were predicted by the calibration curve obtained by use of EMBs for chemical assessment of CIC. In **b**, the predicted CIC values were calculated by use of the calibration curve from Carpenter et al*.* [12], based on a study on formalin-fixed hearts from TM patients with transfusion iron overload (TIO). In **c**, the calibration curve from Wood et al*.* [11] was used, based on dextran-iron loaded Mongolian gerbils. For details see the *Results*. Fat, dotted lines represent lines of equality. **d**–**f** display the agreement between CIC, assessed by use of post-mortem full-wall biopsies, and by CIC values predicted by use of the calibration curve coming from EMBs (**d**), from Carpenter et al. [12] (**e**) and from Wood et al. [11] (**f**). Agreement is assessed by Bland–Altman plots of the mean of 2 methods (x-axis), plotted against their difference (y-axis). The difference is given as absolute figures in mg Fe/g (**d, f**) and as percentages (**g**–**i**). The plots display the bias line (solid line), the 95% confidence lines around the bias, and the 95% confidence lines around the mean difference % (both are dotted lines). For details see the *Results*. Pred. CIC = predicted CIC. FWB = full-wall biopsies

**Fig. 8:**
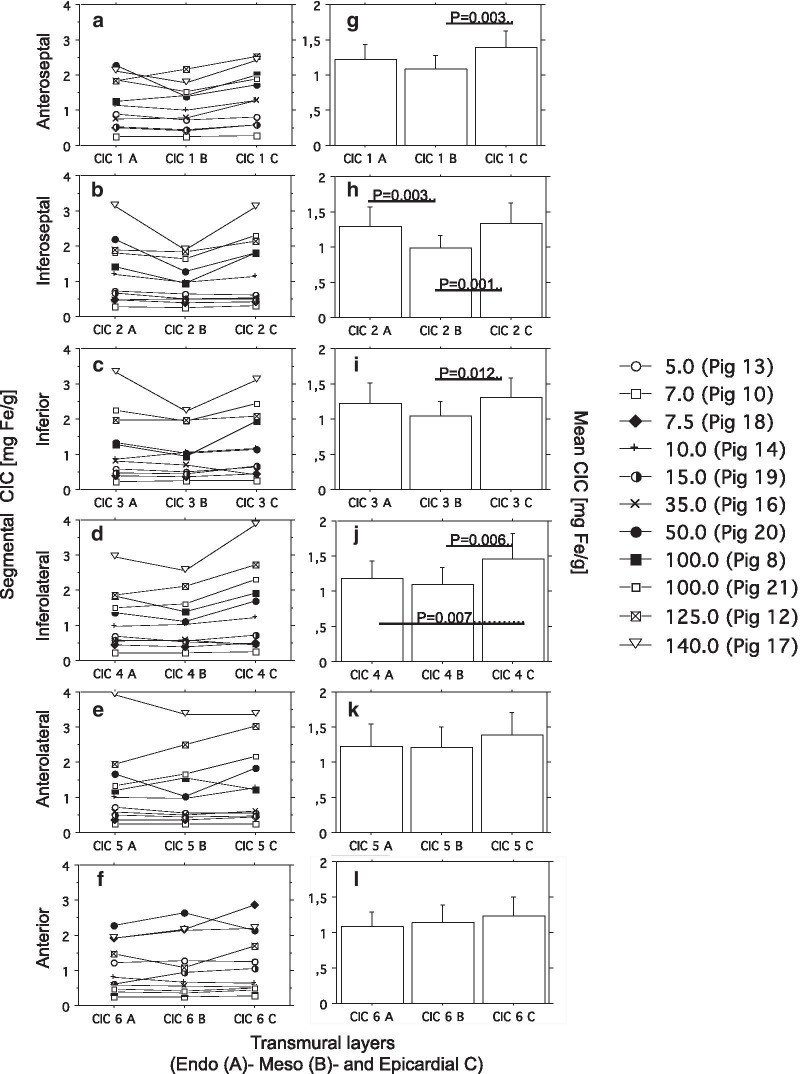
Segmental and transmural distribution of myocardial iron. One mid-myocardial slice was cut from formalin-fixed dextran iron-loaded hearts. Formalin-fixed hearts were available from 11 pigs (7, 8, 12, 13, 14, 16, 17, 18, 19, 20, and 21). The slice was cut into 6 segments: Anteroseptal (1), inferolateral (2), inferior (3), inferoseptal (4), anterolateral (5) and anterior (6). Each section was divided into an epicardial-, mesocardial and endocardial layer, leading to 18 samples from each heart for assessment of chemical CIC (for details see Fig. 2 and the *Results)*. **a** to **f** display segmental and transmural CIC values for each  pig. **g** to **l** display the corresponding mean CIC levels of all 11  pigs for each of the 18 different samples given in mg Fe/g. Significant differences are indicated by horizontal bars and p-values. Label panel gives weekly dextran-iron dose for each  pig in mg Fe/kg BW

**Fig. 9:**
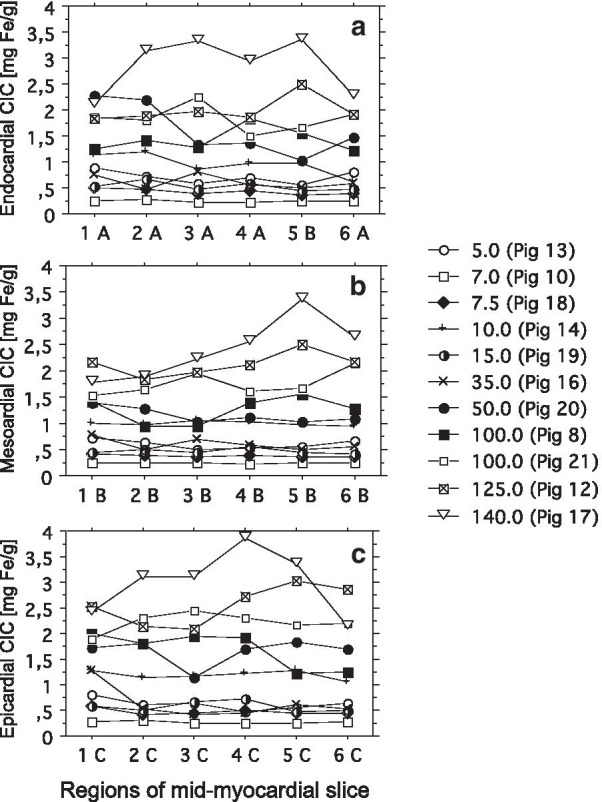
Individual segmental iron distribution investigated in 3 myocardial layers. The individual CIC values are displayed in cell line graphs for the endocardial (**a**), mesocardial (**b**) and the epicardial layer (**c**) for 6 different segmental myocardial regions: Anteroseptal (1), inferolateral (2), inferior (3), inferoseptal (4), anterolateral (5) and anterior (6). CIC values are given as mg Fe/g DW. Label panel gives weekly dextran-iron dose for each pig in mg Fe/g BW

This study includes data from 23 female Göttingen  minipigs. They were obtained from the barrier unit at Ellegaard Göttingen miniswine A/S, Dalmose, Denmark. Animal facilities and principles of laboratory and animal care were as described earlier [21] and conformed to the requirements of the Danish Animal Experiments Inspectorate. The present study was approved by this institution (Approval no. 2013-15-2934-00038) (Figs. 1, 2, 3, 4, 5, 6, 7, 8, 9).

The study comprised 2 groups of  minipigs: Group A contained 11  pigs, which were followed up with CMR for measurement of cardiac T2* and parallel EMBs with 1–3 months intervals for up to 15 months. Ten of these pigs (no. 2 to 11) received weekly intramuscular dextran-iron injection (Uniferon®, 200 mg Fe/ml, Pharmacosmos, Holbaek, Denmark) during follow up.  Pig 1 did not, serving as a control  pig. Group B contained 16  pigs, who had taken a full-wall biopsy from cardiac septum immediately after euthanasia after final CMR session for chemical assessment of CIC. Ten of these pigs (no. 12 to 21) had been dextran-iron loaded from 7 to 21 months and 2 control pigs (22 and 23) had not. Group B also included  pigs 8, 9, 10 and 11 from group A, because full-wall biopsies from cardiac septum, immediately taken after final CMR, were also available from these  pigs. A timeline figure illustrates the dextran-iron dosing over time for the individual pigs in group A and B and the time points of simultaneous CMR and biopsy CMR sessions during follow-up of the pigs in group A (Fig. 1).

The age of the pigs  at final investigation, when the full-wall biopsy was taken, was from 9 to 22 months in group A and from 14 to 28 months in group B, respectively.

Several pigs in the present study had participated in a recently published study on the consequences of long-term dextran-iron loading [21]: Pig 11 in group A (labeled as pig 2 in that study) and pigs 11 to 21 in group B (labeled as pigs 2 to 6, 7 and 9 to 14 in that report).

Principles of iron loading were identical in both pig groups. Intramuscular dextran-iron bolus injection was given weekly, and was dosed by body weight (BW), expressed as mg Fe/kg/week throughout the paper, referring to mg Fe/kg BW/week. One half of the dose was injected into each buttock. When exceeding 5 ml, the bolus was divided into 2 injections on each buttock. The applied doses in group A were 7.0, 7.5, 12.5, 25, 37.5, 50, 75, 100 and 125 mg dextran iron/kg/week and one pig received no iron loading. In group B, the pigs had been iron loaded by 5.0, 7.0, 7.5, 10, 15, 20, 35, 50, 75, 100, 100, 125, 125 and 140 mg Fe/kg/week, respectively. In 2  pigs, the weekly bolus had been increased to 35 mg Fe/kg/week from 5 and 10 mg Fe/kg/week, respectively, after about 12 months of iron loading. Two pigs had not received iron loading. Adjustment of the dextran-iron dose to BW was performed after each CMR/biopsy session. There was always one week between the last dextran-iron injection and the next tissue biopsy/CMR session. After completing the study, the animals were euthanized with an i.v. overdose of pentobarbiturate (Euthasol®, 400 mg/ml, Virbac, Denmark A/S, Kolding, Denmark).

### Anaesthetization of the animals

Before each intramuscular injection of dextran iron, the pigs were anaesthetized with a single dose of tiletamin (Zoletin®0.2 ml/kg, Vibrac). Before CMR scan and invasive procedures with biopsies, the pigs were anaesthetized with repeated doses of tiletamin. The trachea was intubated with a 6.5 mm cuffed endotracheal tube. Before breath-hold during the T2* investigation general anesthesia was achieved by addition of 1.2% sevoflourane (Sevoflourane®, 1 ml/ml, Baxter, Soeborg, Denmark). A dedicated CMR-compatible respirator (Servo ventilator 900c, Siemens, Erlangen, Germany) maintained artificial ventilation (N_2_0:O_2_ mixture was 2:1) between breath-holds during CMR.

### Sampling and procession of EMBs

Right ventricular biopsies were always taken after finished CMR examination. First, under general anesthesia, a central venous catheter (Avanti + 8F, Cordis Corporation, Miami Lakes, Florida, USA) was inserted surgically into the external jugular vein. Myocardial biopsies (6 to 8 fragments in each biopsy session) were taken x-ray-guided by a trained cardiologist with a 7F bioptome (Cordis® Corporation), inserted via the jugular vein to the ventricular septum. Pigs 5–10 from group A, and all group B pigs received a bolus injection of 150 mg amiodarone (Cordarone®, Sanofi Winthrop Industries, Paris, France) before biopsy sampling to prevent arrhythmic episodes.

For assessment of chemical tissue iron, 2 to 3 biopsies were immediately transferred to two 1.5 ml Eppendorf tubes® containing RNA*later®.* The tubes were stored at room temperature for one day. Next day the biopsies were transferred to -20^0^C for long-term storage*.* Storing of biopsies in RNA*later*® was chosen to have the possibility for later gene expression studies on left over biopsy material. CIC was determined by ICP-OES (see below). For later histological examination 2 further biopsies were transferred to another 1.5 ml Eppendorf tube® (Eppendorf AG, Hamburg, Germany) containing 4% formalin solution.

### Sampling and procession of post-mortem full-wall biopsies from cardiac septum

Post-mortem biopsies were taken from the last 4 pigs (8, 9, 10 and 11) entering the present study (group A). As had been performed in  pigs in group B, full-wall biopsies were taken immediately after euthanasia from cardiac septum. For this purpose, we either used a knife, or biopsy needle Bard® Biopsy-Cut® (14 g × 160 mm, Bard, Covington, Georgia, USA). Biopsies were immediately transferred to a 1.5 ml Eppendorf tube® (Eppendorf AG) containing 0.9% isotonic sodium chloride solution for rinsing for blood. Afterwards the biopsy was dried on blotter paper and thereafter transferred to a dry 1.5 ml Eppendorf tube® for storage at − 80 °C. Biopsies for histological examination were transferred to another 1.5 ml Eppendorf tube® containing 4% formalin solution.

### Segmental analysis of the distribution of CIC

Immediately after euthanasia, full-wall biopsies were taken from the ventricular septum of the excised heart. Afterwards the heart was stored in a plastic container containing a 10% neutral buffered formalin solution. Duration of storage was from 1 to 3 years. From 11 pigs (7, 8, 12, 13, 14, 16, 17, 18, 19, 20, 21), the formalin-fixed hearts were available for segmental analysis of CIC. Prior to chemical assessment of CIC, excess formalin was removed. A mid-ventricular slice was cut from the heart and the epicardial fat was removed. Afterwards the slice was cut into 6 segments: Anteroseptal (1), inferolateral (2), inferior (3), inferoseptal (4), anterolateral (5) and anterior (6) (Fig. 2). Each segment comprised the full myocardial wall. Each section was divided into an epicardial-, mesocardial and endocardial layer, leading to 18 samples from each heart for assessment of the segmental, chemical CIC.

### Chemical assessment of cardiac tissue iron concentration

Before measuring the tissue iron concentration, given as mg/g dry weight (DW), the biopsy was dried at 95 °C for 3 days. Immediately afterwards the biopsy was weighed. The sample weight was between 0.24 and 1.88 mg for EMBs, and between 4 and 170 mg for post-mortem full-wall biopsies taken from cardiac septum. A detailed description of the subsequent microwave-assisted acid digestion of the samples, followed by elemental analysis of the digested samples, using inductively coupled plasma-optical emission spectroscopy (ICP-OES) has been given earlier [21]. Three analytical wavelengths were selected for determination of iron (238.204, 239.562 and 259.940 nm) and 2 wavelengths were selected for sulfur (180.731 and 182.034 nm). For analytical quality control, bovine liver (NIST 1577b) standard reference material was digested and analyzed together with the samples. The recovery was within the confidence interval of the consensus values of each element. Correction of chemical CIC values for RNA*later®* content was achieved by subtracting the weight of the RNA*later®* content in the biopsy from the biopsy weight, before calculation of the iron concentration. The RNA*later®* content ((NH_4_)_2_SO4) was calculated as sulfur concentration x biopsy weight × 4.12 (molecule-weight ratio between (NH_4_)_2_SO4 (132.14 g/mol) and sulfur (32.06 g/mol).

### Histological examination of myocardial biopsy sections

Immediately after sampling, myocardial biopsies were formalin fixed, and thereafter paraffin embedded. Then 3–4 µm sections were cut from the blocks. Then, routine staining with Perls’ Prussian blue reaction was undertaken to assess histology and iron deposition in particular.

### CMR examinations for assessment of cardiac T2*

All CMR scans were performed on a clinical 1.5 T CMR scanner (Discovery 450; General Electric Healthcare, Waukesha, Wisconsin, USA) at the Department of Radiology, Aalborg University Hospital, Denmark. The software package of the scanner was version 24. Cardiac T2* was measured by use of a breath-hold, fast gradient multi-echo sequence supplied by General Electric Healthcare). Coil configuration: GE 8 channel HD cardiac phased array. Scan timing: Flip angle 25^0^, TE 1.0 to 14.7 ms, sampling bandwidth ± 125.0 Hz per pixel. Cardiac electrocardiogram (ECG)-triggered gating was used. Scan range: Field of view 36.0 cm, slice thickness 8.0 mm, spacing 0.0 mm and scan matrix 128 × 128 pixels. For calculation of the cardiac T2* the StarMap analysis tool (supplied by GE) was used. For this purpose, a region of interest (ROI) was drawn that encompassed both the endocardial- and epicardial borders of the interventricular septum. All cardiac T2* measurements were performed in triplicate by choosing three separate ROIs to analyze. The average of these replicated measurements is given.

## Statistics

Group means (± standard deviation (SD)) were calculated for all relevant variables. Upper limits of normal (ULN) are calculated as mean + (2 × SD). Follow-up data is presented by the use of line graphs. Relationships between 2 variables are demonstrated by scatter plots. Investigation for normal distribution was tested by the Anderson–Darling test. The strength of the relationship between chemical CIC and cardiac T2* was estimated by calculating the coefficient of determination (R^2^) in normally distributed data, and by calculating the Spearman rank correlation, if data were not normally distributed. Curve fitting was performed by using classical linear regression analysis (least squares approach), if the data were normally distributed. In case of non-normality of data, non-parametric regression analysis was used (Passing and Bablok). The Cusum test for linearity was used to test the applicability of the Passing and Bablok method. Method comparison was performed by using the Bland–Altman plot.

Variation in CIC between 6 myocardial segments and 3 transmural layers within one mid-myocardial cut slice was investigated by a nested mixed effect model. To estimate the overall effect of segment and layer, we formulated a nested mixed effects model with CIC as dependent and layer and segment as fixed effects.  Pig and segment nested under pig were included as random effects. The standard deviation of the difference in percentage between  layers were calculated by the Statistical Delta Method.

To estimate the overall effect of layers within segments, we formulated a nested mixed effects model with CIC as dependent and layer nested under segment as fixed effect. Swine and segment nested under swine were included as random effects. Wald’s tests are used to calculate the P-values for test of no difference between layers. The statistical programming language R, version 4.0.2 and the R-package lme4 were used to estimate the nested mixed effect models.

A value of P < 0.05 was used to define a significant difference.

Spearman rank correlation analysis, Bland–Altman analysis, Passing-Bablok regression analysis, Anderson–Darling test, Cusum test and Wilcoxon’s paired, signed rank test were performed by use of XLSTAT, Live Science for MAC (version 2020. 4.1, Addinsoft, Paris, FR). Other statistical analyses and data handling was performed using StatView (version 5.0 for MAC 1992–1998 SAS Institute Inc., Cary, North Carolina, USA).

## Results

Eleven pigs (group A) were followed with EMBs and parallel CMR for measurement of cardiac T2* within the cardiac septum, repeated with 1–3 months intervals, during iron loading with dextran-iron for up to 15 months. One of these pigs participated in the follow up without iron loading. For each  pig, the dextran-iron dosing over time, time points of simultaneous CMR and biopsy CMR sessions during follow-up, and time of deaths and of euthanasia are illustrated in Fig. 1a.

The accumulated body iron load after ended loading ranged from 9 to 244 g, reflecting 36 to 967 blood units (1 blood unit = 250 mg Fe), respectively. The  pigs remained healthy, and did not develop signs of cardiac disease, such as dyspnea or edema. One pig (no. 2) died shortly after a liver biopsy due to bleeding complications after 6.5 months of iron loading. Three pigs (4, 6, 7) died from complications to the EMB. Pig 4 died of cardiac arrest, preceded by ventricular arrhythmia, after 5 months of iron loading. For this reason, we started using an amiodarone bolus injection, given 10 min before cardiac biopsy sampling, which prevented further arrhythmic events throughout the study. Pigs 6 and 7 died from heart tamponade developed under the cardiac biopsy sampling at 2-months follow up.

Figure 3 shows a photomicrograph, illustrating the developing cardiosiderosis in the cardiac septum during iron loading, for pig 8, which had been dextran-iron loaded weekly by 100 mg Fe/kg for 15 months and had accumulated the largest body iron load of 244 g iron of all  pigs within group A. Already after 2.4 months of iron loading, the first few, tiny iron aggregates were visible in the biopsy section, while chemical CIC was still normal. The most marked increase in iron deposition was seen after 6.7 months, when CIC had increased to 1.43 mg/g. The cardiac iron staining showed more numerous and larger iron aggregates. During further iron loading, CIC seemed to plateau around the level already reached after 6.7 months. Despite this, coalescing to larger iron aggregates was evident, causing more inhomogeneous iron distribution. After completion of iron loading the CIC was increased by 7 times (to 1.31 mg/g).

Figure 4 shows parallel follow-up data of chemical CIC and cardiac R2* (= 1/T2*) from the 11 pigs (group A). The total number of CMR examinations was 60. At five examinations, the catheter biopsy was not taken for various practical reasons, leaving 55 parallel biopsy/CMR investigations. Seven  pigs contributed with 5 to 8 CMR examinations, and 4 pigs with 2 to 4 examinations. Before start of iron loading, mean R2* for all 11  pigs was 25.00 ± 1.60 (SD) Hz, giving an ULN of 28.18 Hz. Mean CIC, expressed in mg/g DW, was 0.15 ± 0.079 (SD), ULN of 0.31 mg/g. When comparing changes in R2* (Fig. 4a) and in CIC (Fig. 4b) during iron loading, the overall pattern of changes was similar:  Pigs, loaded weekly by less than 37.5 mg/kg iron and the control  pig  (no. 1), had R2* and CIC values that remained below ULN, or were only slightly elevated, but still very near the ULN during the whole follow-up period.

Pigs 5, 8, 9 and 11, which had received 50, 100,125 and 75 mg Fe/g BW, respectively, had already raised R2* at first follow up after start of iron loading, and remained so throughout the study, while CIC still was below ULN, except  pig 11. CIC was first elevated at next follow up and remained so until study end (except one measurement in  pig 5). Despite a similar pattern in change of R2* and CIC during iron loading, considerable individual fluctuation and disagreement between curves is evident.

In order to obtain a calibration curve, we investigated the relationship of R2* to CIC (Fig. 5). As the cardiac R2* data and CIC data had been collected as repeated measurements in the same 11  pigs, the individual measurements were not independent. Therefore, the assumptions for using classical linear regression and calculation of the coefficient of determination were not fulfilled. Instead, non-parametric regression analysis was used (Passing and Bablok), requiring a linear relationship. The Cusum test of linearity indicated a linear relationship (P > 0.05). A slope of 41.1 (95% CI 32.5–57.9) and an intercept of 17.2 (95% CI 13.24–19.6) was found. However, marked scatter of data points around the regression line was evident, leading to wide confidence intervals around slope and intercept. Despite this scatter, a close correlation between cardiac R2* and CIC was found (Spearman correlation coefficient rho = 0.72, P < 0.001).

We suspected that the marked spread of data points might be due to variation in CIC, caused by sampling error when taking EMBs. Therefore, we investigated, if measurements of chemical CIC in full-wall biopsies from cardiac septum, taken immediately after euthanasia, in connection with the last CMR session, would give a stronger relationship of cardiac R2* to CIC. Such biopsies were available from 16 dextran-iron loaded  pigs (group B). Dosing over time and duration of dextran-iron loading of each pig is illustrated in Fig. 1b. This relationship was indeed much stronger (R^2^ = 0.98, P < 0.001) and the Spearman rank test indicated a closer correlation (rho = 0.95, P < 0.001).

When assessing the calibration curve (Fig. 6), we used classical linear regression analysis due to normal distribution of the samples (Anderson–Darling test), but also non-parametric regression analysis (Passing and Bablok), in order to enable a comparison with the EMB-based curve, assessed by non-parametric analysis. The curve, obtained by classical linear regression, had a slope of 31.8 (95% CI 29.1–34.6) and an intercept of 17.2 (95% CI 14.3–20.2). Slope and intercept, obtained by non-parametric regression analysis, were not significantly different: slope 31.3 (95% CI 28.2–35.6) and intercept 16.7 (95% CI 14.8–19.7).

Figure 6c displays the same data as in panel a and b, but the individual cardiac R2* measurements are given with error bars (mean of triplicate measurements ± SD). It is obvious that the variation in R2* values increases with increasing CIC.

Figure 7 shows a comparison of the relationships of CIC values, assessed chemically from full-wall septum biopsies, to CIC estimates, predicted by using different calibration curves, investigated by non-parametric regression analysis. Figure 7a shows the relationship between chemically measured CIC values and CIC estimates, predicted by use of the EMB-based calibration curve. The regression line had a slope of 0.72 (95% CI 0.65–0.81) and an intercept of − 0.005 (95% CI − 0.05 to (− 0.07)), which was significantly different from the line of equality. In Fig. 7b, the predicted CIC estimates were calculated by use of the calibration curve from Carpenter et al*.* [12], using the equation [Fe] = 45 × (T2*)^−1.22^, and in Fig. 7c by the calibration curve from Wood et al*.* [11], derived from Fig. 3 in that publication ([Fe] = (R2* − 20)/25.4). Although the CIC estimates, predicted by the last 2 mentioned calibration curves, are close to the line of equality, the slopes of both curves were significantly different from the line of equality. The slope for Wood et al. [11] was 1.23 (95% CI 1.10–1.37) and for Carpenter et al. [12] 0.87 (95% CI 0.76–0.96). The respective intercepts were -0.013 (95% CI − 0.20 to (− 0.007)) and 0.27 (95% CI 0.24–0.35).

The deviation of these 3 regression lines from the line of equality suggested bias between the methods. Therefore, we assessed the bias and also the limits of agreement for the 3 different methods, by comparison with chemical CIC measurements from septal full-wall biopsies, considered as our gold standard, by use of Bland–Altman plots (Fig. 7d–i).

All 3 methods showed a significant bias. The smallest bias of 0.11 mg Fe/g (95% CI 0–0.22 mg Fe/g) was found for the calibration from Wood et al. [11], corresponding to 3.2% (95% CI − 10.4 to 16.7%). The bias was 0.18 mg Fe/g (95% CI 0.11–0.24 mg Fe/g) for the calibration from Carpenter et al. [12], corresponding to 34.1% (95% CI 15.8–52.4%) and for the EMB-based calibration − 0.20 mg Fe/g (95% CI − 0.28 to (− 0.11) mg Fe/g), corresponding to − 23.2%, (95% CI − 33.3 to − 13.2%). The respective limits of agreement for differences were from − 46.7 to 53.0%, from − 33.2 to 101.4% and from − 60.3 to 13.8%. Inspection of the Bland–Altman plots revealed the largest percentage differences around the normal range of CIC (ULN of 0.31 mg/g), indicating lowest method agreement within this CIC range for all 3 methods. We therefore repeated the method comparison after exclusion of the 5 pigs with the lowest CIC (< 0.4 mg Fe/g). This procedure increased the bias for the EMB-based method slightly, from − 23.2 to − 28.8%, but its confidence interval decreased (CI − 35.1 to (− 22.5%)). For the calibration from Carpenter et al. [12], the bias decreased from 34.1 to 13.6%, and had a much smaller confidence interval (CI 4.8–22.6%). The bias for the calibration from Wood et al. [11] increased from 3.2 to 7.7% (CI − 24.4 to 36.7). The limits of agreement indicated better method agreement for all 3 methods: − 47.3 to − 10.4%, − 12.5 to 39.7% and − 24.4 to 36.7%, respectively.

Finally, we investigated the regional and transmural variation in CIC between different myocardial segments by assessing the CIC in 3 transmural layers (epi-, meso-, and endocardial) in 6 different segments (anteroseptal (1), inferoseptal (2), inferior (3), inferolateral (4), anterolateral (5) and anterior (6)) in one mid-myocardial cut slice. CICs were compared by use of a nested mixed effect model. We found a gradient of iron in the transmural layers within the mid-myocardial slice: The CIC was lowest in the mesocardial layer. Within the endocardial layer, CIC was 9.3 ± 3.8% higher (P = 0.015) and within the epicardial layer 19.1 ± 3.5% higher (P < 0.001) than within the mesocardial layer. Moreover, CIC within the epicardial layer was 12.1 ± 3.5% higher (P = 0.007) than within the endocardial layer.

We found no significant differences in CIC between the 6 myocardial segments. When comparing CIC within the 3 transmural layers between the 6 segments (Fig. 8), a the same pattern of a lower mesocardial CIC than endo- and epicardial CIC became evident: Thus, mesocardial CIC was significantly lower than epicardial CIC in all segments apart from the anterolateral and anterior segment, and was lower than the endocardial CIC within the inferoseptal segment. The largest significant difference was found in the inferoseptal segment, where mean CIC in the mesocardial layer was 26.5% lower than in the epicardial layer (P < 0.001). A significantly higher CIC within the epicardial than the endocardial layer was only found within the inferolateral region (19.2%, (P = 0.007). It is evident from panel A to F in Fig. 8, that transmural differences in CIC were absent at normal and slightly elevated CIC values.

Figure 9 illustrates the segmental variation in CIC for each transmural layer for each  pig. By inspection it is obvious, that this variation increases with increasing myocardial iron load in all 3 layers and is most pronounced in the most heavily iron-loaded  pig (no. 17), having received a dose of 140 mg Fe/kg. This pig had markedly higher CIC in the infero- and anterolateral segment than in the 2 septal segments. This variation in segmental and transmural myocardial iron distribution suggested, that a biopsy from the myocardial septum may underestimate the global myocardial iron load of the heart increasingly with increasing myocardial iron load. We therefore investigated the relationship between the CIC, assessed within the anteroseptal segment, and the mean CIC of the other 5 segments of the whole mid-ventricular slice, after calculation of the mean CIC of the 3 transmural layers for each segment. Linear regression (Passing and Bablok) showed a slope, indistinguishable from 1 (1.12, 95% CI 0.92–1.66) and an intercept indistinguishable from 0 (− 0.14, 95% CI − 0.70 to 0.02), data not shown. The Bland–Altman plot revealed a small bias of 0.4% (95% CI − 17.9 to 18.7). Thus, the septal CIC appeared sufficiently representative of the global CIC, at least within the mid-myocardial slice, despite segmental and transmural variation in myocardial iron distribution.

## Discussion

This is the first report on an in vivo follow-up study of chemically assessed CIC during iron loading in an animal model. Such follow-up studies in humans do not exist. Animal follow-up studies of the CIC during iron loading by repeated EMBs are not possible in small-animal models due to the small size of the heart. In contrast, the Göttingen minipig has a heart-to-body weight ratio similar to humans, and also cardiac anatomy, metabolism and electrophysiology is comparable to humans [22].

The heart size allows repeated biopsies from the heart and therefore enables the use of the same animal as its own control. The Göttingen minipig has been recently described as a large-animal model of TIO [21]. Minipigs are increasingly recognized as a suitable non-rodent model and are with increasing frequency used in cardiovascular research, since their anatomic and physiologic similarities to humans favor their use [23].

In the present study, we used follow-up of a small cohort of minipigs by EMB from right cardiac septum during iron loading with dextran iron, in order to get access to iron-loaded myocardial tissue biopsies for chemical assessment of CIC, to be used for calibration of cardiac T2* values, measured in parallel.

The EMB can have serious complications, wherefore it is not used routinely in humans for monitoring cardiac iron. During follow up of 11 pigs, in the present study, 3 pigs died from complications to the EMB, one due to cardiac arrhythmia and the other two to cardiac perforation with tamponade. Compared to the number of performed biopsies (N = 55), the total percentage of fatal biopsies was 6%, and the risk of cardiac tamponade was 4%. This complication rate is higher than observed in a recent, large retrospective study on 175 heart transplanted patients who had 2117 biopsies taken, demonstrating an overall complication rate of only 0.7%, including one patient with cardiac tamponade [24]. In another, earlier, prospective study, including 546 EMB sessions, an overall complication rate of 6% was found [25]. This study included patients with a serious cardiac state and a high average number of collected specimens (6 ± 2 fragments). In the present study we usually sampled 6 to 8 fragments in each biopsy session, in order to get sufficient tissue for CIC assessment and other examinations, which might explain the apparently high frequency of cardiac tamponade. Development of cardiac arrhythmias, including ventricular fibrillation with little provocation, is a recognized problem in pigs [23]. For this reason, we started using prophylactic amiodarone injection before catheter biopsy, after having lost one pig due to ventricular fibrillation during biopsy sampling. This approach prevented further arrhythmic episodes.

The main goal of the present study was, by use of the minipig model of TIO, to construct a calibration curve from CIC values, measured chemically from serial EMBs, and from parallel cardiac T2* measurements. Our study shows that this is possible during iron loading in minipigs, receiving different dextran-iron loading rates. Spread of CIC values within the range of CIC values, intended to be calibrated, was obtained by investigating the pig at different timepoints during iron loading, in the present study up to 8 times. By using this approach, a calibration curve could be achieved, despite keeping the number of investigated animals small. This is in contrast to small-animal models, e.g. the Mongolian gerbil, where each animal only can be investigated once because it has to be euthanized before biopsy sampling.

Cardiac R2* was strongly, linearly correlated with CIC, leading to [CIC] = (R2* − 17.16)/41.12 for the calibration equation with CIC in mg/g DW and R2* in Hz. The relationship, demonstrated between CIC and cardiac R2*, was closer than it might be expected, when acknowledging that the EMB has been described as unreliable for measuring cardiac iron, due to large sampling variation [5, 6]. Although the EMB sampling is X-ray guided, it is somewhat blind, and may lead to accidental sampling from structures containing less or no iron deposits. Accidental sampling outside the right ventricular septum may also affect the measured CIC due to a possible regional variation of iron deposits, as observed in human iron-loaded hearts [5, 12, 20, 26, 27]. Also, biopsies, sampled from the right ventricular septum, may contain different amounts of connective tissue from the subendocardial layer, and especially very small biopsies may be expected to contain less or no iron at all. Moreover, a transmural iron gradient in the septum, with lesser iron deposits in the endocardial than the mesocardial part of the septum, as demonstrated in iron-loaded human hearts [12, 28], may contribute to larger variation in CIC values, caused by variation in depths when sampling biopsies from the septal wall by the catheter, compared to post-mortem full-wall biopsies.

As expected, we found, that EMB-based CIC measurements had lower levels than CIC measurements from full-wall biopsies. We found a significant bias of around − 23%, leading to a systematic underestimation of the septal CIC when using EMB.

In order to explain this difference, we re-analyzed the CIC in hearts from 11 pigs, that had been stored in formalin after sampling the full-wall biopsies and performed differential measurements of endo- meso- and epicardial CIC in a mid-myocardial cut slice. In contrast to our expectations, CIC within the endomyocardial layer of the right ventricular septum was not significantly lower than within the other 2 layers. This finding suggests that the lower CIC in EMB-based measurements is likely to be a consequence of a proportionally larger content of less iron-containing connective tissue from the subendocardial layer in EMB than in full-wall biopsies.

An underestimation of the septal iron concentration by EMB may also explain, why parallel follow up of cardiac T2* and chemical CIC during dextran-iron loading of the miniswine revealed that R2* in cardiac septum increased before CIC. This finding suggests that the endocardial layer of the right ventricular cardiac septum, that is accessible by the catheter biopsy, is iron loaded later than the remaining part. Therefore, measurement of cardiac T2* should be more sensitive for diagnosing early cardiosiderosis than EMB.

Our finding of a much closer relationship, when using the larger full-wall biopsies, instead of EMBs, supports the notion that small sample size and sampling error is an important issue when assessing CIC from EMBs. Therefore, when using EMB for assessment of cardiac iron, either in the clinical setting or in future calibration studies of CMR-derived iron estimates, this risk of sampling error should be taken into account.

A transmural iron gradient, as in the present study, has also been observed in the left ventricular myocardium in the study of Carpenter et al. [12] on 12 severely iron-loaded human hearts: In the most heavily iron-loaded heart, the CIC was highest in the epicardial layer, intermediate in the endocardial and lowest in the mesocardial layer, while in all other hearts, at mean, the epicardial CIC was significantly higher than the endocardial, and the mesocardial was not significantly different from the other 2 layers. In contrast, in our iron-loaded  pigs, a significantly higher CIC within the epicardial, than the endocardial, was only seen in the inferolateral region, while a lowest iron level within the mesocardium was seen in all segments, except the anterior and anterolateral segment. Thus, minipigs also develop uneven transmural iron distribution during dextran-iron loading, similar to the findings in human iron-loaded hearts caused by TIO, but the uneven distribution appears to develop at lower myocardial iron levels in the minipigs hearts than in human hearts. The reason for the transmural gradient is unknown.

The linear correlation between R2* and CIC was very strong when using full-wall biopsies (R^2^ = 0.98). The relationship was even stronger than in the study of Carpenter et al*.* [12] (R^2^ = 0.91) and in that of Wood et al*.* [11] (R^2^ = 0.94), although the range of CIC values (independent variable) was larger in both studies, favoring a large R^2^ value. The use of fresh cardiac tissue, biopsy sampling without sampling error, lack of motion artefacts due to anaesthetization of the animal are some of other possible explanations for the stronger relationship.

Visually judged, the calibration curve of Carpenter et al*.* [12] was very close to the line of equality. However, when comparing CIC values, predicted by that calibration curve, with measurements from full-wall septum biopsies from the miniswine, the slope was significantly different from the line of equality, and there was a significant bias of 34.11% (13.61% after exclusion of swine with CIC around the normal range). As the calibration curve from Carpenter et al. [12] is based on human hearts, and our calibration curve comes from minipig hearts, the bias prohibits the use of the pig -derived calibration curve for direct translation of human cardiac R2* values to CIC estimates. The bias should be subtracted. Alternatively, the equation from Fig. 7b, CIC (Carpenter) = 0.27 + 0.87CIC (minipigs), obtained from regression analysis of the CIC estimates, predicted by use of the calibration curve from Carpenter et al. [12], on CIC measurements from porcine full-wall septum biopsies, might be an applicable correction equation. The use of the corrected pig-derived calibration should, however, be restricted to cardiac R2* values outside the normal range, due to poor method agreement around the normal range. The reason for the poor method agreement around the normal CIC range is unclear. The studies of Carpenter et al. [10] and Wood et al. [11] did not include this CIC range. In an earlier study, we found that mean myocardial R2* in the unloaded minipigs (25.98 ± 1.60 Hz) is very near the findings in normal human hearts (27.5 ± 1.5 Hz, [29], as well as ULN of chemical CIC in the unloaded  minipigs (0.31 mg/g DW) is also near that in humans (0.47 mg/g DW [30].

This conformity of cardiac T2* and CIC in normal humans and normal minipigs on one hand, and the finding of a systematic, but correctable difference between CIC estimates, based on the calibration from human hearts (Carpenter et al. [12]), and chemically CIC values obtained from full-wall biopsies from the iron-loaded  pig hearts, suggest that the bias is a consequence of different effects of myocardial iron loading on myocardial R2* in the pig hearts and the human heart. Iron deposition in the myocardium during iron loading, caused by blood transfusions, is different than by iron loading with intramuscular dextran-iron treatment. Thus, in the minipig model, cardiac iron loading is predominantly in the myocardial RE cells, and iron loading within myocytes is only seen at high rates of iron loading [21]. This pattern of cardiac iron loading is also described in dextran-iron loaded rodents [31]. In contrast, e.g. in patients with thalassaemia major with TIO, cardiac iron loading is predominantly within myocytes [32]. The storage of iron in different cell types may lead to different iron distribution at the cellular scale, influencing the relative strength of changes in T2 and T2* caused by iron loading. Although T2* has been shown to be significantly more robust to changes in intercellular and intracellular iron distribution than T2 [33, 34], a significant bias was demonstrated, likely caused by different changes in relaxation properties during myocardial iron loading in the human and in the minipig hearts.

An explanation of the bias by formalin fixation of the hearts in the study of Carpenter et al. [12] is less likely, because, according to that study, formalin fixation of the hearts caused only very little variation of T2* over time. This findings is also in accordance with an earlier study [35]. As the bias is correctable, the minipig  model of TIO may be a useful substitute for human cardiac tissue samples for calibration studies of cardiac T2* and also for development of iron-sensitive CMR sequences in the future.

The increase in individual SD of mean R2* measurements at increasing CIC illustrates an increasing amount of variation in cardiac R2* with increasing myocardial iron deposits. Likely, this is caused by an increase in heterogeneous iron deposition across different myocardial regions, across the myocardial wall, and probably also by change in iron distribution at cellular level as well. Increase in SD by increasing CIC widens the confidence interval of the calibration line. Multiple measurement in separate ROIs within the cardiac septum are therefore advisable at increasing myocardial iron loading when using the iron-loaded minipig heart for calibration studies.

The most important advantage of using minipigs for calibration is the easy access to biopsies. In the study of Carpenter et al*.* [12], it took seven years to gather 12 iron-loaded human hearts. In the minipig model useful cardiac iron deposition can be achieved within a half to one year or even faster, if larger iron-loading rates of dextran iron are used, than in the present study. By using different iron-loading rates, a range of CIC values of clinical relevance can be covered.

Another important advantage, compared to the Mongolian Gerbil model and other small-animal models of TIO is, that the minipig represents a large-animal model. As aforementioned, the heart of the  minipig has human dimensions, allowing the use of clinical CMR machines without hardware constraints imposed by using a clinical scanner for scanning of small animals. Thus, cardiac T2* measurements can be performed at higher quality by using identical sequence parameters as those used for humans, including the image scaling factor, voxel size and slice thickness that may influence T2*. Moreover, the heart rate in minipigs is about the same as in humans, in contrast to the high heart rate found in Mongolian gerbils, allowing assessment of cardiac T2* by multi-echo imaging instead of by use of single-echo/gradient-echo sequences, as necessary in gerbils [11].

### Limitations

The most important limitation, when using the minipig model for calibration of cardiac T2*, is that the calibration cannot be directly transferred to human hearts. This is due to the demonstrated bias between the CIC, assessed in the porcine septum by chemical analysis, and predicted CIC estimates, obtained by use of a calibration curve, calibrated with human iron-loaded hearts by Carpenter et al. [12]. The bias is most likely caused by different changes in relaxation properties during myocardial iron loading in the human and in the  minipig hearts due to different distribution of the deposited iron. Therefore, the calibration curve cannot directly be used to translate human cardiac R2* values to human CIC values without an appropriate correction.

Another important limitation is, that it was not possible to calibrate the entire range of clinically relevant CIC values, due to the resistance of the  minipig myocardium to iron loading. Thus, cardiac iron loading during dextran-iron loading appears later and requires several-fold larger body iron loads to achieve clinically relevant CIC values than in gerbils and in TM patients with TIO [21]. Accordingly, the range of chemical CIC values obtained from full-wall biopsies in the present study is narrower compared to the two other mentioned calibration studies [11, 12]. The range in the present study was 0.15 to 2.0 mg/g DW. In the study of Carpenter et al*.* [12], the range was 3.2 to 9.5 mg/g DW and in the study of Wood et al*.* [11] 1.3 to 6.6 mg/g DW (calculated from Figure 3 in Wood et al*.* [11]). Thus, the present minipig study calibrates a range of T2* values that has not been calibrated by the 2 other studies. Although this calibration covers a clinically important CIC range, that is encountered in heavily transfused non-thalassaemic patients, as for e.g. in patients with myelodysplastic syndromes [14], a wider calibrated CIC range had been desirable. A higher iron-loading rate, than used in the present study, may possibly have yielded higher CIC values, but due to the mentioned resistance of the minipig against cardiac iron loading, it is not likely that as high CIC values as in gerbils and TM patients with TIO would be achievable. This would require too large dextran-iron doses, impossible to administer safely by intramuscular injections.

Finally, the sample size in the present study is only just adequate. A larger sample size would have been desirable, a specially when assessing method agreement.

## Conclusion

Calibration of CMR-derived cardiac T2* for estimation of CIC by EMBs from cardiac septum is possible in the minipig model, but it is less accurate than with full-wall post-mortem septum biopsies, likely because of sampling error, variable content of non-iron containing tissue and smaller size of biopsies when using the catheter biopsies.

The presented results further validate the T2* technique for estimation of cardiac iron in conditions with iron overload and add to the limited calibration data published earlier.

The  minipig model of TIO will also be of interest for development and calibration of new CMR sequences for estimation of CIC, and in the future, the model may replace calibration with cardiac samples from patients with TIO.

## Data Availability

The datasets generated during and/or analyzed during the current study are available from the corresponding author on reasonable request.

## Abbreviations

BW: Body weight
CIC: Cardiac iron concentration
CMR: Cardiovascular magnetic resonance
DW: Dry weight
ECG: Electrocardiogram
EMB: Endomyocardial catheter biopsy
ICP: Inductively coupled plasma spectrometry
ROI: Region of interest
TIO: Transfusional iron overload
ULN: Upper limit of normal
